# Obituary for Yuri Dubrova (1955–2023): Germ cell mutagenesis pioneer

**DOI:** 10.1093/mutage/gead008

**Published:** 2023-03-22

**Authors:** Carole Yauk

**Affiliations:** Department of Biology, University of Ottawa, Ottawa, ON, Canada


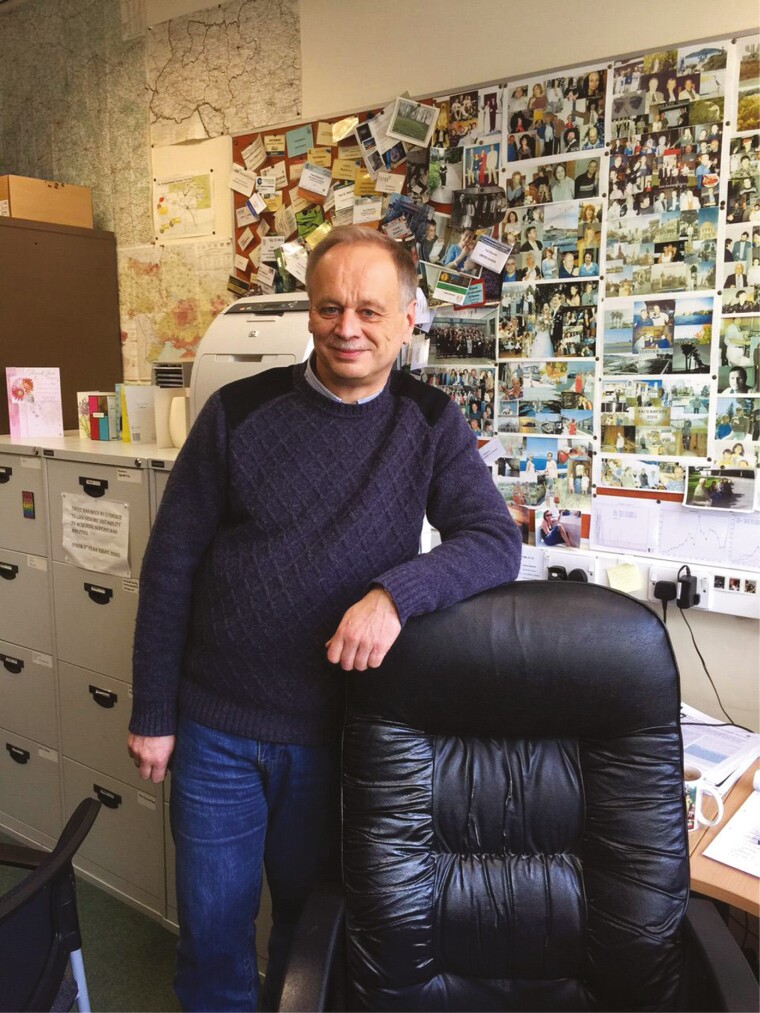


Professor Yuri Dubrova’s research transformed our understanding of environmental mediators of heritable genetic effects. His discovery that the tools and loci used in DNA forensic sciences could be repurposed to study induced mutagenesis opened the door to research investigating the relationship between environmental exposures and germ cell mutations in species across kingdoms. His provocative findings that low-dose radiation exposures in humans are associated with increased rates of heritable germ cell mutations set the stage for contentious debates around the world. He has undoubtedly left an indelible mark on the field of mutation research.

Dubrova was born in Kyiv, Ukraine, in 1955. He obtained a BSc in Biology at Kiev State University followed by a PhD in Genetics at N.I. Vavilov Institute of General Genetics in Moscow. During his PhD, he studied under the renowned Yuri Petrovich Altukhov, head of the Laboratory of Population Genetics at the Institute of General Genetics, who was leading research to demonstrate that environmental exposures, including radiation, cause heritable genetic effects [1]. This work challenged findings on the lack of heritable mutagenic effects in survivors of acute radiation exposures in Japan following the atomic bombs in Hiroshima and Nagasaki, as consistently reported by the Atomic Bomb Casualty Commission. In this contentious training ground, Dubrova expanded his knowledge of population genetics and germ cell mutagenesis, setting the stage for his innovative research program that courageously challenged conventional thinking.

Dubrova’s curiosity was piqued by the minisatellite DNA fingerprinting technology invented by Professor Sir Alec Jeffreys in the 1980s. Dubrova hypothesized that the hypervariable loci underlying DNA fingerprinting would be exquisitely sensitive to induced mutagenesis due to their high baseline mutation rate. He forged a collaboration with Jeffreys that ultimately led him to accept a Royal Society Visiting Research Fellowship and uproot his family to join the Department of Genetics at the University of Leicester in the UK in 1991. He remained at the University of Leicester for the duration of his career, progressing to become a Wellcome Trust fellow, lecturer, and later a Professor of Genetics. Below is a summary of a few of his exceptional breakthroughs, which many of us heard for the first time at Environmental Mutagenesis and Genomics Society meetings, that greatly influenced an entire generation of germ cell mutagenesis scientists.

Dubrova was deeply committed to understanding the ramifications of exposure to radioactive contaminants on population health. His early years at the University of Leicester led to seminal papers that gained immediate ­critical ­international attention. Working alongside Jeffreys, Dubrova used DNA fingerprinting to study tandem repetitive elements of the mouse genome to ask whether these could serve as neutral biomarkers of male-mediated heritable mutagenesis [2]. At this time, the gold standard to study germ cell mutagenesis in mice was the Mouse Specific Locus test invented by William L. Russell [3], which required high-dose exposures and up to 100,000 mice to quantify mutations associated with seven phenotypic traits [4]. In humans, population genetic studies focussed on phenotypic effects (e.g. birth defects) or 2D protein electrophoresis to differentiate structural changes in protein profiles in offspring relative to their parents. These technologies lacked the sensitivity and power (requiring tens of thousands of individuals) to detect subtle changes in low environmental mutagen exposures.

At Leicester, Dubrova showed that acute exposure of male mice to relatively low doses of radiation (0.5 or 1 Gy-γ-radiation) caused an increase in tandem repeat mutations in their offspring that could be measured using DNA fingerprinting [2]. With a sample of 232 offspring collected from 26 irradiated and control families, he found a significant increase in heritable mutations in the irradiated families, with a doubling dose consistent with values derived from the Mouse Specific Locus Test. Because all eukaryotic organisms possess similar highly unstable repetitive sequences in their genomes, the findings armed Dubrova with the empirical evidence needed to support extending this research to humans and other organisms exposed to environmental sources of radiation.

Dubrova first assessed minisatellite DNA fingerprints in humans to determine if nuclear fallout from the Chernobyl nuclear plant disaster in Ukraine would have implications to the unexposed descendants of people inhabiting these contaminated locations. Never shy of controversy, Dubrova published his alarming results in *Nature* [5] where he reported a doubling in inherited germline minisatellite mutations in the offspring of people inhabiting heavily polluted areas of the Mogilev district of Belarus after the Chernobyl accident relative to a control population. These findings set the stage for historic debates with key figures in the field for years to come [6]. Dubrova strove to tackle each criticism in turn, returning to the laboratory to continuously build on his story by, for example, addressing confounders and demonstrating a dose-response [7], advancing the evidence supporting that the increased mutation rates were due to exposure to radioactive contamination. He later expanded his studies to include sites of former nuclear weapons testing [8] and provided weight-of-evidence to support the work by demonstrating impacts in other sentinel species, including wheat [9]. In this way, his research reignited interest in the study of environmentally induced germ cell mutagenesis.

In parallel with his research in humans and the induction of transmissible *de novo* mutations through the exposed germline, Dubrova hypothesized that the indirect effects of radiation exposure could ultimately lead to persistent genetic instability, manifested as transgenerational increases in mutation rates. Toward this, he made the remarkable discovery that the F2 descendants of irradiated male mice continued to exhibit an increase in tandem repeat mutation rate despite being a generation removed from the exposure [10]. He extended the work to show that the genetic instability could be observed in somatic cells and identified effects occurring in other genetic endpoints including *Hprt* mutations [11]. Together, these findings provided compelling evidence that epigenetic effects induced by radiation may mediate persistent genetic instability that can affect future generations, catalyzing a new area of research.

Dubrova accomplished many more things in his career that are too extensive to detail. For example, he expanded his studies beyond radiation to demonstrate similar effects manifested after exposure to chemical mutagens, including anti-cancer drugs [12]. In the genomics era, he applied new sequencing technologies to decipher the spectrum of heritable genomic changes caused by radiation. He showed that *de novo* copy number variants, insertion/deletion events (indels), and a novel type of clustered mutations are significantly elevated in offspring of irradiated male mice [13]. The work offered intriguing mechanistic insights into the efficacy of lesion repair and the diverse mutational landscape induced by radiation, providing a compelling argument to pursue such work with other germ cell mutagens.

Dubrova was known for much more than his science. He trained many post-docs and PhD students and left a legacy of passionate scientists in his footsteps. He had an incredible sense of humour, a wholehearted love for the arts, and a true zest for life. He will be widely remembered for his larger-than-life personality and presence at meetings, his passion for the field, and his linguistically colourful endorsements of what he appreciated scientifically and socially. He will be sadly missed by his family, colleagues, trainees, and members of the Association for Radiation Research and the Environmental Mutagenesis and Genomics Societies.
